# Fracture of Radius1Read before the Missouri Valley Association, at Kansas City, January 13th.

**Published:** 1896-03

**Authors:** L. M. Klutts

**Affiliations:** Clinton, Mo.


### FRACTURE OF RADIUS.[1]

BY DR. L. M. KLUTTS,
CLINTON, MO.

THE subject, Prince, a favorite surrey horse. On arriving, I found my patient standing on three legs, with the fourth dangling in the air. By a careful diagnosis, I found the radius

[1] Read before the Missouri Valley Association, at Kansas City, January 13th.

had received a complex fracture about three inches above the knee, which had received such a shock that the superior row of bones seemed to move as if out of place. On manipulation I could distinctly hear crepitation of a number of pieces of detached bones. The capsular ligament of the knee was undoubtedly ruptured, and the synovia had caused that soft, puffy feeling that we so frequently have in bog-spavin, and a few other slight bruises, completed my external diagnosis. Haggard looks, fits of shivering, and quick jerking of the body indicated excruciating pain internally.

The horse ran in front of a train until he came to the guard; then jumped, and got hung between the ties, and the engine came along, struck him and knocked him against the tie with such force as to break it in two and tumble him down the embankment. I told the owner perhaps it would be a humane act to destroy him; although, if he thought best, we would take him over to his house and give him a chance for his life, to which he readily consented. We forced him to hop on three legs four blocks to the stable.

I got plaster-Paris, oakum, and material for bandages, and elevated the fractured limb about four inches, letting the hoof rest on a box, set the leg, and had an assistant hold the leg in position. I saturated the oakum in a rather thin liquid of plaster and water, applied it next to the skin, then built my plaster-Paris cast firmly around the fracture. I only used one splint, and that I fastened on behind the leg securely to the cast. I enclosed the cast and splint in a dry bandage, and turned him loose. In a very short time he lay down, being very nervous from pain, and we allowed him to lie for an hour or so. When he got up he was allowed the privilege to run loose in a paddock. I directed them to feed him on bran, oats, and good hay, and to be careful to watch him, as he would be liable to get down on the side of the fractured limb, and he would have to be turned over before he could get up.

I visited him from time to time and found the local fever and swelling gradually disappearing, until about the ninth day, I found him very restless. He lay down on the fractured leg, and in struggling to get up wrenched it pretty bad, so much so that we soon found more swelling and fever than ever. I braced up my cast and turned him loose the second time, thinking in a few days we would have suppuration from the injury, and the result would be death; but instead the swelling and fever disappeared, and he being decidedly more careful never to lie down on that side any more until he could use it enough to get up. In three weeks I removed the cast, and I was agreeably surprised to find the bones all united nicely, but considerably enlarged, but this has gradually reduced until the leg is almost the size of the opposite one.

I have had most excellent success treating fractures in the horse and other domesticated animals in simpler forms, but this is the only time I ever had a complex fracture complicated with dislocation of a joint and rupture of capsular ligament of same to come out so well.

The chances for recovery of fractures are more favorable in good-dispositioned animals.

I seldom use slings, plaster-Paris cast, leather shield, bandages, and splints; decision always concluded on examination and finding out the surrounding circumstamces, generally to turn them loose in a large box-stall or paddock, where they are free from other stock.

CASE II. Subject, Johnnie C., a blooded horse. On arriving I made an examination and found an incision about eight inches long in the abdominal region just about four inches from the anterior part of the sheath, and slightly to one side, the result of jumping on a picket and post. The small intestines had protruded and were held up by means of a bandage made from a horse-blanket, which the trainer had when the accident happened; this kept the intestines from becoming entangled in his feet until something could be done. I found the mesentery slightly lacerated and the intestines considerably congested and my patient reeling and staggering from exhaustion and pain, and had that well-marked deathly gloss in the eyes. With a bucket of warm water and a bed of nice, clean straw, which fortunately was near at hand, before I could apply hobbles to lay him down he lay down from weakness, and the assistants fastened him. I applied warm water and sponged off the dust and straw from the intestines, and proceeded to return them, loop after loop. At first I found my task exceedingly tedious on account of the distention, and thought of what a practitioner had told me a few years ago, who had a similar case, and, it being impossible to return them, he decided to enlarge the incision, and the result was, instead of a bucketful, he had a tubful and lots more ready to come. From the result of this conversation I concluded not to enlarge the incision. I finally returned the last loop, and while I had my assistant hold them in, I prepared to stitch up the incision. I trimmed off the ragged edges of the incision and brought them together with braided silk No. 4. I made the regular surgeon's loop-stitch the entire length of the incision. I then stitched up the incision in the skin, applied a mild anodyne liniment around the wound and let him up, sent him to his stall, a distance of eight blocks, where I found him next morning, to my surprise, living and eating hay, the local injury but little swollen. But little suppuration followed, which healed by first intention. He did not miss a feed, and was soon ready to go out and work.

